# Role of MALDI-MSI in combination with 3D tissue models for early stage efficacy and safety testing of drugs and toxicants

**DOI:** 10.1080/14789450.2021.1876568

**Published:** 2021-02-02

**Authors:** Chloe E Spencer, Lucy E Flint, Catherine J Duckett, Laura M Cole, Neil Cross, David P Smith, Malcolm R Clench

**Affiliations:** Centre for Mass Spectrometry Imaging, Biomolecular Sciences Research Centre, Sheffield Hallam University, Sheffield, UK

**Keywords:** Mass spectrometry imaging (MSI), organoids, GI tract

## Abstract

**Introduction**: Three-dimensional (3D) cell cultures have become increasingly important materials to investigate biological processes and drug efficacy and toxicity. The ability of 3D cultures to mimic the physiology of primary tissues and organs in the human body enables further insight into cellular behavior and is hence highly desirable in early-stage drug development. Analyzing the spatial distribution of drug compounds and endogenous molecules provides an insight into the efficacy of a drug whilst simultaneously giving information on biological responses.

**Areas Covered**: In this review we will examine the main 3D cell culture systems employed and applications, which describe their integration with mass spectrometry imaging (MSI).

**Expert Opinion**: MSI is a powerful technique that can map a vast range of molecules simultaneously in tissues without the addition of labels that can provide insights into the efficacy and safety of a new drug. The combination of MSI and 3D cell cultures has emerged as a promising tool in early-stage drug analysis. However, the most common administration route for pharmaceutical drugs is via oral delivery. The use of MSI in combination with models of the GI tract is an area that has been little explored to date, the reasons for this are discussed.

## 1. Introduction

The pharmaceutical industry is currently worth in excess of a trillion US dollars and this value is continuously rising [1]. Numerous dosage forms have been designed and enhanced for pharmaceutical drugs that vary in administration route: namely oral, topical, and injection. One of the most important challenges during drug development is to determine if the administration route/dosage formulation delivers the drug to the required site of action. Visualizing the spatial distribution of endogenous and exogenous molecules within tissues provides vital information about how a therapeutic penetrates, distributes, and metabolizes. It can in addition allow the observation of biological responses to the treatment.

Conventional methodologies to localize molecules in tissue samples or *in vivo* include immunofluorescence microscopy [2], positron emission tomography (PET) [3], and magnetic resonance imaging (MRI) [4]. However, these methodologies have limitations such as requirements for the addition of fluorescent labels, or magnetic or isotopic probes. However, addition of such a probe can have an effect on therapeutic pathways or can alter biological compositions, and additionally such probes have limited use with human subjects. Mass spectrometry imaging (MSI) is an established method which can simultaneously map a variety of molecules within a tissue, without the use of labels. MSI applications extend to a range of ionization techniques including, but not limited to, matrix-assisted laser desorption ionization (MALDI), desorption electrospray ionization (DESI), and liquid extraction surface analysis (LESA). MALDI-MSI is the most widely used technique with high spatial resolution and increasing speed of acquisition, which make it an appealing analytical method for high-throughput drug development studies. The multiplex nature of MALDI-MSI also enables the analysis of different tissue types and biological models for quantitative applications detecting a range of molecules including metabolites, lipids, peptides, and proteins [5–8].

The combination of MALDI-MSI with animal models has been demonstrated in numerous drug studies [9–13], with the first MS images of pharmaceutical detection achieved in mouse tumor and rat brain tissues in 2003 [14]. The use of animals in research has become prominent for drug development due to their complex biological environment that provides a more advanced *in vivo* representation, modeling specific aspects of human diseases. Animals are therefore considered the ‘gold standard’ model system for the evaluation of new therapeutic approaches in cancer and disease biology. For MALDI-MSI, methods of handling and preparing animal specimens have been established, from freezing and sectioning tissues to matrix application [15–17]. Therefore, this methodology is highly validated for pharmaceutical analysis. Progressive research has, however, challenged whether animal studies are an appropriate model to predict human responses [18]. It is strongly argued that the failure of animal models to replicate human conditions contributes to the failure of the majority of therapeutics at clinical trials [19,20]. Further challenges are also raised regarding the regulatory, economic, and societal issues with the use of animal models involved [21].

There is high demand for alternative biological models that accurately replicate the *in vivo* environment and responds to the societal requirements to reduce animal numbers in research. Three-dimensional (3D) cell cultures are an advanced system that bridges the gap between two-dimensional (2D) cultures and animal models. Such an approach enhances the structural complexity of cellular cultures so that they more closely mimic the *in vivo* microenvironment of primary tissues. These 3D models promote levels of cell differentiation and tissue organization, which replicate typical tumor characteristics of gene and protein expression, nutrient diffusion, and cell-cell and cell-matrix interactions [22]. A variety of 3D culture models have been developed to meet the biological requirements for specific research including drug analysis [23], patient-derived treatment [24], and biological crosstalk [25]. These models include spheroids, organoids, and microfluidic systems or ‘organ-on-a-chip’. Each model varies in their levels of complexity and yet requires relatively low maintenance to achieve representative *in vivo* qualities. With the additional advantages of low cost and high throughput, the use of 3D models is appealing for early-stage drug research and development prior to *in vivo* studies.

Studies which combine MSI with 3D cell culture models are currently of considerable interest, especially in the fields of drug efficacy and toxicity. The current literature in these areas is discussed within this review and is summarized in Table 1. The future potential for MSI analysis of ‘up and coming’ *in vitro* models is also discussed, identifying a gap in research regarding the oral delivery administration route and modeling of the gastrointestinal (GI) tract. Oral delivery is the most popular route for administration amongst patients, and therefore its popularity also extends to researchers. However, analyzing the oral delivery route presents many challenges due to the complexity of studying drug absorption in the GI tract. The enclosed physiology and fluctuating conditions experienced in the GI tract make the accurate study of oral drug absorption complex, which consequently hinders the progress of drug development [26]. From the progress of MALDI-MSI analysis with 3D models, and the developments of modeling the GI tract, it seems this combination is the next suitable direction for early-stage drug efficacy and toxicity studies.

**Table 1.** Table includes a summary of literature reporting the analysis of 3D culture models with MSI discussed within review. Table is not limited to all publications of 3D culture models in combination with MSI

Paper	Year Published	Disease	3D Model	3D Method	MSI Method	Spatial Resolution	Summary
Li and Hummon. *Anal. Chem*. [30]	2011	*HCT 116 colon carcinoma*	Spheroids	Aggregation	MALDI	75 µm	MSI detected specific regional distributions of proteins across the 3D culture structures and highlighted a central necrotic region. Proteins including Histone H4 and cytochrome C identified in spheroids, validated by LC MS/MS.
Ahlf Wheatcraft, Liu and Hummon. *J. Vis. Exp*. [31]	2014	*HCT 116 colon carcinoma*	Spheroids	Aggregation	MALDI	unreported	Spheroid-MSI workflow protocol. Includes the culture, preparation and analysis of spheroid samples by MALDI-MSI.
Hiraide *et al.*, *Sci. Rep*. [32]	2016	HCT 116 & DLD-1 colon carcinoma. Cancer tissue-originated spheroids (CTOs) – primary cells.	Spheroids	Aggregation followed by Cellmatrix type I-A scaffold gel	(AP) MALDI	5 µm laser spot size	Tandem MSI identified specific lipid species arachidonic acid containing PI (18:0/20:4) within the outer region in spheroid culture. Phospholipid associated with metastatic behavior.
Liu, Weaver and Hummon. *Anal Chem*. [33]	2013	HCT 116 colon carcinoma	Spheroids	Aggregation	MALDI	75 µm	Drug penetration of irinotecan mapped over a 72 h time-dependent course, moving from outer region of spheroid into the core. Detection of drug metabolites within spheroid validated by nanoLC MS/MS.
Li and Hummon. *Sci. Rep*. [34]	2016	HCT 116 colon carcinoma	Spheroids	Aggregation	MALDI	75 µm	MSI of platinum-based drugs and their metabolites displayed heterogeneous distributions within spheroids by derivatization. UPLC MS/MS quantification.
Lukowski, Weaver and Hummon. *Sci. Rep*. [35]	2017	HCT 116 colon carcinoma	Spheroids	Aggregation	MALDI	75 µm	Demonstration of doxorubicin-encased liposomes delivery into spheroids. Detection of doxorubicin and active metabolites throughout spheroid by 72 h.
Feist *et al., Anal. Chem*. [36]	2017	HCT 116 colon carcinoma	Spheroids	Aggregation	MALDI	75 µm	Proteomic imaging of histone peptides and their posttranslational modifications determined the response to epigenetic drug within serial sections of spheroids, generating samples from core, the mid and external areas.
LaBonia *et al., Anal. Chem*. [37]	*2018*	HCT 116 colon carcinoma	Spheroids	Aggregation	MALDI	75 µm	Proteomic changes from a dynamic flow dosing with FOLFIRI treatment investigated and quantified by iTRAQ analysis. Heterogeneous distribution of drugs and active metabolites throughout spheroid.
Liu *et al., Anal. Chem*. [38]	2018	HT-29 & DLD-1 colon carcinoma Colorectal cancer	Spheroids Cancer organoids	Aggregation Patient biopsies	MALDI	70 µm	On-tissue reduction and alkylation method to detect time-dependent penetration and distribution of monoclonal antibody, Cetuximab. Different localizations of immuno-drug determined between two cell lines. Detection in organoid cultures also demonstrated.
Flint *et al., Anal Chem*. [43]	2020	HCT 116 colon carcinoma	“Aggregoid”	Alginate scaffold followed by aggregation	DESI, imaging mass cytometry, LA-ICP-MSI	30 µm 1 µm 6 µm (laser spot)	Characterization of a novel aggregated spheroid model by metabolites, proteins and metals determined phenotypic regions of the tumor microenvironment for pre-clinical applications.
Avery *et al., Xenobiotica* [47]	2011	Healthy human epidermal skin	Skin organoid	Straticell-RHE-EPI-001 Reconstruct-ed Human Epidermis	MALDI	150 µm	Demonstrated a MALDI-MSI method of an artificial skin model analysis by detection of imipramine absorption.
Francese *et al., Anal. Chem*. [48]	2013	Healthy living skin equivalent (LSE)	Skin organoid	Evocutis “LabSkin”	MALDI	50 µm	The evaluation of curcumin as a matrix for MALDI across different applications, lung tissue, artificial skin and fingermark. Lipid imaging identified epidermis and dermis regions within the LSE. Detection of acitretin distribution within LSE.
Harvey *et al., Proteomics* [49]	2016	Healthy and psoriatic LSE	Skin organoid	Innovenn “Labskin”	MALDI	100 μm	Absorption of acitretin within psoriatic LSE by MALDI-MSI determined a larger depth of penetration in psoriatic model compared to healthy LSE.
Russo *et al., Anal. Chem*. [50]	2018	Healthy living skin equivalent (LSE)	Skin organoid	Innovenn “Labskin”	MALDI	60 μm	Quantitation of terbinafine hydrochloride absorption into epidermal region of LSE by MALDI-MSI. Regions of LSE identified by detection of abundant lipid species. Penetration enhancer DMI increased the concentration of drug. Validated by LC MS/MS.
Bergmann *et al., Nat. Proto*. [52]	2018	Blood-brain-barrier (BBB)	Organoid	Co-culture of endothelial cells, pericytes and astrocytes by low-adhesion conditions.	MALDI	unreported	Development of a BBB organoid platform for drug analysis by fluorescent microscopy and MALDI MSI. Analysis of BKM120 and dabrafenib permeability with BBB organoids.
Johnson *et al., J. Mass Spec*. [53]	2020	Pancreatic cancer	Cancer organoid	Patient biopsies	MALDI Orbitrap	75 μm	Method development of organoid cultures into geltain microarrays for high-throughput MSI analysis. Organized array of organoids in the same z-axis for multi-sample imaging.
Liu *et al., J. Am. Soc. Mass Spectrom*. [41]	2018	Colorectal cancer	Cancer organoid	Patient biopsies	MALDI	35 μm	Heterogeneous distribution of irinotecan and its active metabolites within organoid cultures demonstrated metabolic variability in complex tissue samples.
David *et al., ACS Medicinal Chemistry Letters*. [42]	2018	MCF-7 breast cancer	Cancer organoid	Mice xenograft	MALDI TOF	unreported	*Ex vivo* method of visualizing peptides and small molecules in breast cancer tumor explants as an alternative to spheroid cultures. Detection of small molecule drug (4-hydroxytamoxifen) and peptide drug (cyclosporin A) as proof-of-concept.

## 2. Types of 3D cell culture models studied by MSI

### 2.1. Tumor spheroids

Tumor spheroids have become essential tools for *in vitro* research due to their ability to replicate the *in vivo* microenvironment. These tumor models blend the flexibility of cell culture systems with the ability to assume complex cellular architecture displaying a hypoxic gradient that can be divided into three regions: a necrotic core, which experiences a high rate of apoptosis due to the extremely poor delivery of oxygen and nutrients; a non-proliferative region, where the cells display a state of dormancy as a result of hypoxia; and a proliferative edge with an abundant supply of nutrients, which accelerate tumor growth. The creation of spheroids can be achieved by a variety of means, either through independent culture or co-culture with different cell lines, followed by aggregation [27]. Additionally, the use of scaffolds [28] or culturing with embedding gels [29] may be incorporated into the model.

The Hummon group were the first to publish work describing the combination of MSI with spheroids and have continued leading research utilizing spheroid cultures with MSI for drug analysis. Their initial study developed a colon carcinoma spheroid culture from the HCT 116 cell line. Li and Hummon [30] adapted previous MALDI-MSI protocols for imaging tissue sections, to examine the protein distribution within spheroids. To assist the handling of tumor spheroids, the group embedded the samples within gelatin prior to flash freezing and cryo-sectioning tissues at a thickness of 10 µm. A protocol describing the workflow of tumor spheroids with MALDI-MSI was published by group [31]. From the study, protein images of the spheroids were obtained in positive mode at a spatial resolution of 75 µm. MALDI-MSI was able to detect species within specific regions of a spheroid; with the majority of peaks distributed across the section, and a specific unidentified peak at *m/z* 12,828 localized predominantly within the central necrotic region. The individual peaks detected were not identified directly from the MSI data. Alternatively, the group employed an in-gel tryptic digest of the spheroids and identified species, including Histone H4 and Cytochrome C, by MALDI profiling and liquid chromatography tandem mass spectrometry (LC MS/MS), correlating the *m/z* values to the MSI ion maps. The detection of species localized within specific regions of the spheroid identified phenotypic differences that corresponded to the hypoxic gradient, therefore MALDI-MSI enabled a further understanding of the model. This was demonstrated by another study from Hiraide *et al*. [32], who utilized atmospheric pressure (AP) MALDI-MSI to characterize lipids throughout spheroids and determined the species that are specific to cancerous tissues. The group used an MS/MS imaging approach to identify *m/z* 885.5 as an arachidonic acid-containing phospholipid PI (18:0/20:4) specifically accumulated in the outer edge of a colorectal cancer model. It was suggested this phospholipid was associated with the migration of cancer cells, which thus identified the species as a potential biomarker for metastatic colorectal cancer. The identification of species in addition to its spatial distribution not only provides important information for understanding the 3D model, but also holds great value for drug distribution studies. This is with regard to determining therapeutic targets, and the penetration and localization of a drug.

The combination of spheroids with MSI has further progressed for drug developmental research. Liu *et al*. [33] investigated the efficacy of drug penetration by analyzing the spatial distribution of irinotecan, an anticancer drug, and topoisomerase I inhibitor, over a time-dependent course within HCT 116 colon carcinoma spheroids. Using MALDI-MSI irinotecan was observed within the necrotic core only after 12 h of incubation. At 24 h, appreciably higher levels of the parent drug were observed within the central hypoxic region and necrotic core, and higher levels of irinotecan metabolites: SN-38, SN-38 glucuronide, and a decarboxylation metabolite were located within the outer region. This indicated that there was a higher activity of metabolizing enzymes in the proliferative outer layer of the spheroid. In addition, high levels of the decarboxylated metabolite were observed by MALDI-MSI and these data were validated by nanoLC MS/MS; however quantitative analysis was not conducted. Using MALDI-MSI the group therefore successfully demonstrated how MSI can be used to locate a parent drug and its metabolites, identifying regions of metabolic activity within a spheroid model. This is highly advantageous for drug efficacy and toxicity studies for screening drugs, determining their targets and their half-life. The Hummon group has further exploited this technique to study the penetration of a range of drugs including platinum-based [34], epigenetic-targeting [35], and liposomal delivery [36]. LaBonia *et al*. [37] recently investigated the penetration of a combinational drug, FOLFIRI (folinic acid, 5-fluorouracil, and irinotecan), within HCT 116 spheroids by an innovative dosing platform to mimic the dynamic flow of chemotherapeutics used *in vivo*. Similar to the previous study high levels of metabolites within the outer proliferative region were observed with parent drugs, irinotecan, and folinic acid, localized within the necrotic core. Proteomic changes to the FOLFIRI treatment were determined by iTRAQ proteomic analysis. Protein-originated peptides labeled with iTRAQ tags allowed for identification of proteins in addition to quantitative fold changes between treated and untreated samples, however spatial resolution within the spheroids was lost. This study provided evidence that MALDI-MSI can detect drug therapeutics and their metabolites within the different regions of the spheroid, alongside the proteomic response to treatment. Not only does this support the proposition that spheroids display regions within the structure that mimics the tumor microenvironment, but it also gives valuable insights into the true behavior of a drug treatment in *in vivo* conditions, which could predict clinical outcome.

From small molecule chemotherapy to immunotherapy, the Hummon group studied HT 29 and DLD-1 colon cancer spheroid cultures and patient-derived organoids to observe the distribution of cetuximab by MALDI-MSI [38]. Biopharmaceuticals are one of the leading growth sectors of the pharma industry, therefore the ability to analyze these complex molecules within tissues is in great demand. There are many challenges experienced when protein imaging (~150 kDa), this is partly due to the low ionization efficiencies and reduced sensitivity [39]. Liu *et al*. [38] developed a direct on-tissue reduction with DTT followed by alkylation to overcome these issues. With this method, a protein is reduced to break the disulfide bonds and alkylated to prevent re-formation by modifying the cysteine residues. Cetuximab was therefore detected using a signal arising from the light chain variable domain at *m/z* 23,412.5. The group observed a difference in the distribution of the light chain domain between the HT 29 and DLD-1 spheroid models; at 72 h cetuximab was primarily localized within the core of the HT 29 spheroids, whereas in the DLD-1 spheroids it was detected in the outer region. It was confirmed by immunofluorescent staining that this was due to the different expression levels of the antibody target, epidermal growth factor receptor (EGFR) in both cell lines. The light chain domain of cetuximab was also detected within colorectal-tumor organoids at 72 h; however, information about the distribution of the antibody within the organoid section was not given. In addition, the study examined the treatment response by detecting higher intensity signals of ATP (*m/z* 506.0) in the core of the HT 29 spheroids, indicating an increase in apoptosis in the presence of cetuximab. Overall, the study provided proof-of-concept that MALDI-MSI has the capabilities to detect the presence of a complex biopharmaceutical (150 kDa) within an emerging 3D *in vitro* model, the tumor spheroid, and analyze the cellular response to treatment.

It is clear from the literature that the combination of spheroids with MSI is a powerful tool to investigate the biological behavior of replicate *in vitro* tissues and study the efficacy of therapeutic drugs. Although there are a range of applications demonstrated, there are still gaps within the literature. As discussed, the main spheroid culture investigated using MSI has been cell-aggregated colon cancer cell lines. MALDI-MSI has great potential to study drug delivery in other cancer types such as breast or lung spheroids, which have been utilized in other experiments [23,40]. In addition, spheroids of co-cultured cell lines would provide an extra level of complexity and thus possibly give data of greater clinical relevance. As previously mentioned, there are various types of spheroids models such as those made within a biomimetic hydrogel scaffold, which acts to recapitulate the behavior of a natural extracellular matrix (ECM). An MSI experiment with these spheroid types could potentially provide information about drug behavior and biological crosstalk within the ECM, which is essential for certain tissue types that grow within a filamentous structure *in vivo*.

**Figure 1.**
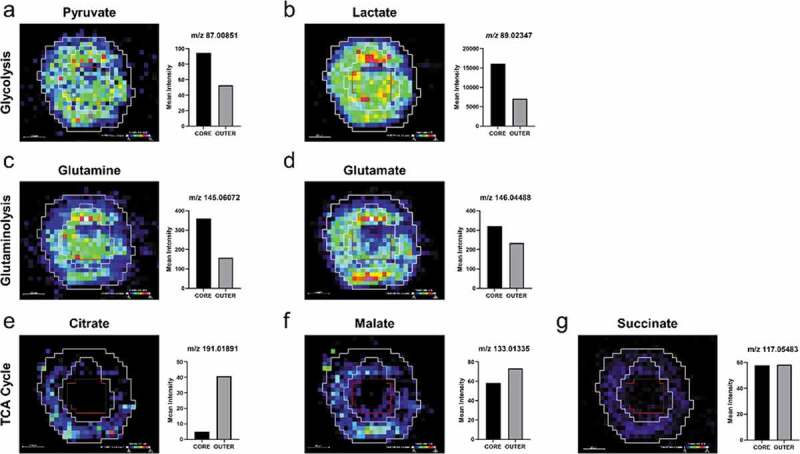
Distribution of metabolites regulating cancer growth and survival within the HCC827 lung adenocarcinoma aggregoid central section by DESI-MSI. Ion density maps of metabolites outlining the core and the outer regions of the aggregoid on the image to highlight hypoxic and proliferative areas, respectively. Mean intensity plotted on bar graph against the core and outer regions. Scale bar 200 μm. Intermediates of the glycolysis reaction: (a) pyruvate, *m/z* 87.00880 and (b) lactate, *m/z* 89.02440. Glutaminolysis reaction: (c) glutamine, *m/z* 145.06190 and (d) glutamate, *m/z* 146.04590. TCA cycle: (e) citrate, *m/z* 191.01980; (f) malate, *m/z* 133.01430; and (g) succinate, *m/z* 117.01940. [Flint *et al*., 2020, Reference [43]]

It is argued, however, that spheroid cultures are unable to fully recapitulate the morphological, phenotypic, and genetic heterogeneity of *in vivo* tumors [41]. This is in part due to the spherical shape they adopt, which does not necessarily capture the complex phenotypical structures observed in patient tumors, impacting how the drug behaves and penetrates the system. It has also been noted that spheroids of certain cell lines of some tumor types, e.g., breast cancer, can be difficult to grow large in size (<100 µm) and prove challenging for MSI to generate an image with sufficient raster spots to observe the substructure [42]. However, the continuous developments in MSI spatial resolution are achievable efforts to overcome this challenge. Although, it is an understandable requirement to use more advanced models that can be grown large enough to study the spatial distribution of molecules. For example, a recent study by Flint *et al*. [43] reported the use of multimodal MSI techniques: DESI, imaging mass cytometry (IMC) and laser ablation inductively coupled plasma (LA-ICP)-MS to characterize a novel aggregated 3D culture model of lung adenocarcinoma. The *in vitro* model, termed ‘aggregoid’ is formed via the aggregation of clonal tumor spheroids to create a more heterogeneous tissue of approximately 1 mm diameter. The molecular information of metabolites, proteins, and metal isotopes from the MSI techniques achieved at great spatial resolution had a complementary nature which enabled an in-depth understanding of the tumor microenvironment and the biological processes within the tissue model (Figure 1). The imaging analysis of the aggregoid demonstrated a potential methodology for drug efficacy and toxicity studies within a complex tumor spheroid model that is more morphologically representative.

### 2.2. Organoids

Derived from patient stem cells or biopsies, organoids are small-scale constructs that adopt the morphological structures of *in vivo* tumors and organs. Like spheroids, these self-organized systems allow for the study of biological processes including cell behavior, tissue repair, and drug response. As organoids are derived from patients, these systems hold the potential to assist in the prediction of drug response in a personalized manner. The first reported organoid structure dates back to 1975 by Rheinwald and Green [44], who cultivated a living skin replacement from epidermal keratinocytes which was later used to treat burn patients. Although organoids first received interest back in the 1970s, within the last decade organoids have witnessed a revival. These systems have been used in a range of studies to investigate healthy and diseased organs or the behaviors of primary tumors to drug treatment. For example, Dye *et al*. [45], generated lung organoids from human pluripotent stem cells and observed their remarkable similarities to human fetal lungs, thus stating it an excellent model for human lung development, maturation, and disease studies. As a study conducted by Crespo *et al*. [46] developed colonic organoids to observe the blocking effects of a chemotherapeutic, geneticin in hyperproliferation, which has been associated with colon cancer.

Since its first development, the human skin organoid has been successfully commercialized by a number of businesses for experimental use. These include: a human reconstructed epidermis (HRE) and a 3D differentiated epidermis culture derived from human keratinocytes known as EpiSkin (Epskin, Lyon, France) and EpiDerm (Mattek, Ashland, USA), as well as full thickness living skin equivalents (LSE) that is T-skin (Episkin, Lyon, France) and Labskin (Labskin UK Ltd, York, UK). The combination of human skin models and MALDI-MSI has been extensively utilized to study the drug absorption into the different layers of the skin. The benefits of using the skin models with MALDI-MSI analysis are that the skin can be treated similarly to animal tissues in terms of sample preparation and imaging acquisition. The earliest work of this approach was conducted by Avery *et al*. [47], who examined the absorption of an antidepressant drug, imipramine into a model of the human epidermis, ‘Straticell’. A considerable difference in the intensities of imipramine at 2 h and 8 h was observed, with higher signals of the drug in the epidermis at 8 h. No quantifiable data analysis was conducted to achieve a significant concentration; however, this is partly due to the lack of quantitative methodologies for MALDI-MSI data at the time the study was conducted. Although this study stated some data remained inconclusive and additional experimentation is required to determine biotransformation of the drug. Avery *et al*., however, clearly understood the potential capabilities of MALDI-MSI to determine drug penetration and its metabolizing properties.

Since this study, the analysis of LSE models by MALDI-MSI has been developed further for a range of drug developmental experiments. Francese *et al*. [48] utilized the skin model to investigate the effects of curcumin as a matrix to promote analyte ionization, with the purpose of achieving efficient detection of the drug acitretin in an LSE model. After 4 h incubation, successful penetration of acitretin within the epidermis was observed by MALDI-MSI at *m/z* 326.4, in addition to analysis of several lipid species. The detected peaks from curcumin were compared to the peaks from the conventional matrix CHCA. Higher intensities with curcumin for both the drug and endogenous lipid species were observed indicating the assistance of analyte ionization. This study demonstrated curcumin as an extremely efficient matrix, which is vital to promote the higher intensities of the drug in tissues for effective detection. The following study by Harvey *et al*. [49] examined the absorption of the same drug, acitretin in psoriasis induced LSE model at longer incubation periods. It was demonstrated by MALDI-MSI acitretin penetrated into the epidermis at 24 h then further infiltrated the dermal layer of the skin after 48 h. To confirm the location of the drug in the LSE model a sodiated sphingomyelin at *m/z* 725.4, which correlated to the epidermis was detected. This time-dependent approach allowed for the evaluation of the drug’s penetration and stability properties within a living tissue. MALDI-MSI successfully demonstrated the capabilities of analyzing drug delivery, whilst also identifying specific regions of a complex organoid skin equivalent model.

**Figure 2.**
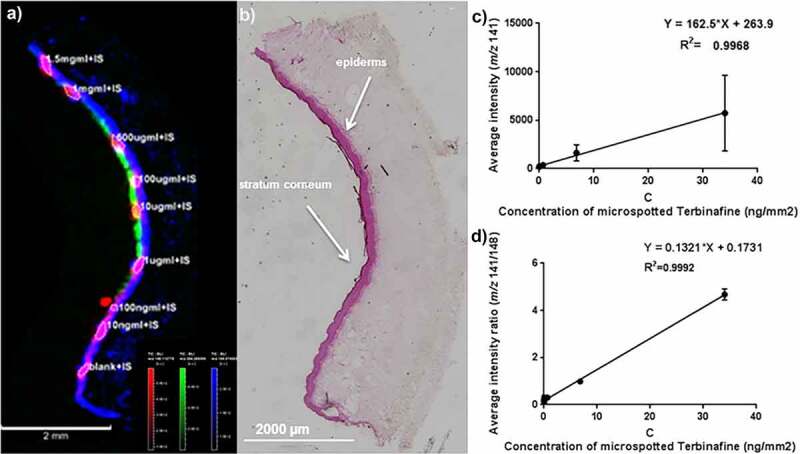
(a) MALDI-MSI of the deuterated Terbinafine (Terbinafine-d7) source generated fragment ion in red (*m/z* 148) superimposed with choline headgroup in blue (*m/z* 184) and ceramide fragment peak in green (*m/z* 264). (b) Hematoxylin & eosin stained optical image of the sublimated section after MALDI-MSI (4× magnification). Calibration curve (n = 3) generated using (c) the average intensity of *m/z* 141 (no normalization) and (d) the ratio average intensity of *m/z* 141/148. Normalization to the internal standard *m/z* 148 improved the linearity of the calibration curve. [Russo *et al*., 2019, Reference [50]]

The advanced technique to localize a drug within an intricate model of the human skin has been further exploited to achieve absolute quantification by MALDI-MSI. A study conducted by Russo *et al*. [50] developed a quantitative MSI (QMSI) approach to determine the amount of an antifungal agent, Terbinafine hydrochloride within the epidermis of the LSE model, ‘Labskin’. The study optimized a micro-spotting methodology to achieve precise and uniform analytical and internal standards at nanolitre volumes solely within the epidermal layer, in order to mimic the cell-type ionization response in treated tissues. Increasing concentrations of the internal standard were successfully detected at *m/z* 148 by MALDI-MSI to generate a calibration curve. Signals from the regions of interest observed endogenous species that defined the epidermis (PC at *m/z* 184) and stratum corneum (*m/z* 264) to ensure the drug calibration signals extracted were true to the specified region (Figure 2). In addition, the study evaluated the performance of the penetration enhancer Dimethyl Isosorbide (DMI) added to the delivery formulation. QMSI detected an increase in concentration of Terbinafine with an increase in percentage of DMI within the epidermis of the LSE. Validation analysis observed no statistical significance between the values from QMSI and the values from LC MS/MS, thus proving MALDI-MSI as a powerful quantitative method. This study demonstrated the potential impact QMSI with tissue engineered models will have on drug development. By determining the amount of drug present within a tissue, information of its pharmacological activity can be obtained, in addition to observing ion suppression effects across varying tissues or regions within the same tissue.

The combination of MSI with tumor organoids is a relatively new approach. More conventional imaging techniques such as fluorescence microscopy have mainly been used to observe these tumor models [51]. However, efforts of MSI strategies to analyze tumor organoids have been reported, either in combination with fluorescent microscopy to detect the penetration of small molecule drugs that are inherently non-fluorescent [52], or the development of sample preparation methods to improve high-throughput analysis [53]. Tumor organoids are similar in size to tumor spheroids, and therefore require embedding medium, such as gelatin, to assist sample handling prior to preparation for MSI analysis following conventional protocols. Liu *et al*. [41] reported the use of MALDI-MSI with patient-derived colorectal tumor organoids (CTOs) to observe the drug distribution of irinotecan in a time-dependent dosage. MALDI-MSI detected high intensities of irinotecan at *m/z* 587.3 and its metabolites SN-38 (*m/z* 393.1) and SN-38 glucuronide (*m/z* 569.2) were distributed differently within the CTOs at 24 h of dosage. It was stated that this was possibly due to the various cell types including ISCs, differentiated enterocytes, goblet cells, entero-endocrine cells, and Paneth cells that form the organ model, which could have metabolized irinotecan differently. Thus, supports the benefits of utilizing organoids over single-cell type spheroids to understand the metabolism of therapeutics within a structure comprised many cell-types. The study also employed a QMSI approach to determine the amount of irinotecan present in the CTOs compared to its metabolites at a higher dosage at 72 h, observing lower signal of SN-38 and indicating less conversion of the active metabolite from irinotecan. This approach proved a valuable insight in the capabilities of QMSI to determine drug efficacy and potential chemo-resistance in patient-derived CTOs, which in turn could predict clinical outcome that is specific to the patient.

Alternative organoid cultures that mimic *in vivo* tissues other than skin or colorectal tumors have yet to be studied by MSI. Various challenges of cell culturing or MALDI-MSI ionization may be within optimization; however, there is progress toward the development of the MSI-organoid approach. For example, a study conducted by David *et al*. [42] developed a method to study tumor explants of breast cancer xenografts. MALDI-MSI successfully monitored the distribution of macrocyclic peptides and small molecule treatments *ex vivo* in mice tumors. Although the group utilized mouse tissue, the ability to extract biopsies and detect the uptake of therapeutics demonstrates a promising analytical method to examine patient-specific treatment for prevalent diseases such as breast cancer. Based on the current success, imaging analysis of complex organoid cultures has led to the ability to spatially locate and quantify drugs. This emerging technique is proving extremely valuable and it will be interesting to see where organoid imaging can take drug developmental research further.

Recent developments of cutting-edge technology have enabled further advancements with organoid cultures. Three-dimensional bioprinting enables fabrication of highly complex multi-cellular tissues by combining cells, growth factors, and biomimetic materials. This technology has revolutionized tissue engineering due to its versatile processing capabilities to recapitulate important structural features of functional organs, which in the long term could be considered for transplantable tissue in regenerative medicine [40]. Bioprinting also has the potential to be used for personalized medicine for cancer treatment. For example, Zhao *et al*. [41] constructed an *in vitro* cervical cancer model by the fabrication of HeLa cells with hydrogel-based materials, observing a significant difference in chemo-resistance to the anti-cancer drug, paclitaxel, compared to 2D cell culture. Evaluation of anti-cancer treatment using 3D bioprinting is still a relatively new concept, however it has the potential to be used as a pre-clinical *in vitro* study tool in drug development. MSI has not been used to exploit 3D bioprinting to the best of our knowledge to date, however it seems that this could in future be used as a valuable tool for *in vitro* drug analysis.

## 3. Future potential

As previously mentioned, the most common route for drug delivery is orally [54,55]. Oral drugs are highly convenient for patients and allow them to self-administer drugs without the assistance of a trained professional or the need for a visit to a hospital or clinic [54–56]. This route also boasts a high patient compliance, undoubtedly related to the painless and relatively noninvasive nature of this route in comparison to others such as intravenous (IV) injection, for instance [55,57]. Despite this, the physiologically enclosed route creates difficult challenges for drug developers and researchers. The oral administration route is a complex system to replicate, with several hollow organs that serve different purposes and therefore, vary in structure and environment [58,59]. Differences in environment exhibited within the GI tract include extreme fluctuation and variation in pH when moving through different organs and the presence of acidic gastric juices [59,60]. Varying gastric emptying rates and phenomena such as the first-pass metabolism are also observed in the GI tract; all of these factors must be considered when creating models to represent the GI system as they have a substantial effect on the delivery and absorption of oral drugs [59,60]. Additionally, some drug classes also face degradation from the digestive enzymes present in the GI tract [57,60]. In order to develop and approve pharmaceutical drugs, the effect of the fluctuating environments and challenges presented by the GI tract on an oral drug must be greatly studied to observe and fully understand how their delivery will be effected prior to administration to a human patient. In the drug discovery and pre-clinical stages of drug development, human trials are prohibited without extensive data relating to drug safety as regulated by the Medicines and Healthcare products Regulatory Agency (MHRA) [61]. This highlights the need for reliable models of human physiology and specifically for orally administered drugs, reliable GI models with adequate means of quantifying the drug absorbed.

### 3.1. Modeling the GI Tract

The absolute gold standard for reliable oral drug studies would be to conduct *in vivo* studies on human volunteers. *In vivo* refers to an experiment which has taken place within an entire living organism. The most common practice for oral studies is to perform mass balance experiments which involve dosing the live participants with radiolabelled drugs [58,59]. The purpose of mass balance studies is ultimately to establish the absorption level of oral drugs in either human or animal participants [58,59]. The amount of radioactivity of excreta is quantified and compared to the radioactivity within the original drug dosage [59]. The quantification of drug molecules identifiable with a radiolabel is reported to be relatively simple considering the complex biological environments in which it is found [59]. The detection and quantification of drug molecules are classically established using a combination of liquid chromatography with radioactivity detection and on-line MS detection [62]. LC-MS is currently widely used for the detection, identification, and quantitation of drug metabolites from such mass balance studies [62]. The benefit of this particular mass spectrometry method provides the utmost accurate and sensitive quantitative information from an *in vivo* sample, giving true to life results.

Although as previously mentioned, this type of experiment would be prohibited in drug discovery and pre-clinical stages of drug development. It is also hindered by extortionate expenses relating to radiolabels and the price of using human volunteers. Such studies could be conducted on animal subjects, however as previously stated, this immediately creates an additional disadvantage to the technique in terms of the ethicality of this practice and the societal issues regarding the use of animals. In the current world, the proposal and consequent enforcement of the Directive 76/768/EEC which oversees the ban of using animals for testing cosmetic products and ingredients have undoubtedly influenced some research groups in drug-related fields to move toward an animal-free practice [21]. Currently, there are several different approaches and models available that have been produced and developed for the study of drug absorption in the GI tract; they each have their own purposes and unique advantages, and limitations that are addressed by other models [54,58]. As a result, drug development tends to utilize all of these approaches at different stages of oral drug development [58]. These techniques include *in situ* perfusion, *in-silico* models, and *in vitro* methods. Here we evaluate the application of mass spectrometry techniques such models.

#### 3.1.1. In situ perfusion

*In situ* perfusion is a technique that is performed within rats or humans whereby the subject is anesthetized, and their intestinal segments are cannulated and perfused with a drug solution [63,64]. The amount of drug absorbed is then calculated by establishing the difference between the inlet and outlet drug concentration [64,65]. The quantification methods that are routinely used in combination with *in situ* perfusion include LC-MS/MS [63]. Intestinal perfusion within human subjects is typically implemented in the later stages of drug development due to issues with drug safety. As with *in vivo* studies, *in situ* perfusion shares the issue with obtaining human participants for these studies [63].

*In-situ* perfusion of rats is typically used in pre-clinical drug studies and is considered the next best approach to *in vivo* studies due to the maintenance of the natural physiological structure and function of the GI system [66,67]. Ruiz-Picazo and colleagues [68] conducted a study using high-performance liquid chromatography (HPLC) to demonstrate the comparability of data obtained from their *in situ* perfusion rat model known as Doluisio. Using HPLC, the group were able to conclude that Doluisio was a reliable tool for predicting human permeability in the jejunal segment of the GI tract and the colon [68]. Other research groups such as Kim *et al*. [69], compared the regional absorption of the oral antihypertensive agent, fimasartan, in a conventional *in situ* single-pass perfusion method with an improved *in situ* model. The quantitative assessment was made and compared using LC-MS/MS in multiple reaction monitoring mode (MRM) mode. The LC-MS/MS method applied by Kim and their research group was able to demonstrate that the improved *in situ* model gave a more accurate assessment of the fraction fimasartan that had been absorbed [69].

A benefit of performing *in situ* perfusion with rats is the ability to implement imaging techniques on the tissue after the perfusion has occurred which is not possible with humans due to the need for animal sacrifice. After *in situ* perfusion, drug distribution patterns are commonly imaged using techniques such as PET and autoradiography [62]. There is a clear benefit to imaging tissue from drug absorption experiments; with focus on visualizing the distribution of the drug and such, evaluating whether a drug has reached its target. Despite this, the MSI methodology has not been utilized with tissue from *in situ* perfusion experiments with regard to oral drug absorption studies. Addressing the advantages of using *in situ* perfusion models which include the ability to use biological barriers identical to those found in the *in vivo* environment, it would presumably make for an interesting and useful study [66,67]. To conduct a study combining this model with for example MALDI-MSI, additional steps including cryo-sectioning the tissue and matrix application are conventional ways in which it would prepare the previously perfused tissue for analysis via MALDI-MSI.

A major limitation of *in situ* perfusion studies is the use of esthesia which is more invasive and of higher risk than *in vivo* mass balance studies. As consequence of this, considerably more funding is required to make the study more attractive to volunteers. Another downfall to the use of anesthesia, human, or animal is that when combined with surgical manipulation, it could have a significant effect on drug absorption rate [54,67]. It is essential to know how relevant an alternative model is to the *in vivo* situation, whether it be *in situ* or *in vitro* [64]. It is therefore important to establish an *in vitro in vivo* correlation [58].

#### 3.1.2. In Silico models

*In vitro in vivo* correlation (IVIVC) is a mathematical model that is used to predict and describe the relationship between *in vitro* studies and the *in vivo* response [26]. An *in silico* approach is commonly used to obtain an IVIVC in oral drug studies [59,67,70]. In this instance, *in-silico* refers to computerized models which are used to simulate the drug absorption process within the GI tract. There are different *in-silico* models available ranging in complexity and their typical uses; these include, but are not limited to, the quantitative structure-activity relationship (QSAR) model and physiologically based pharmacokinetic (PBPK) modeling [59,67].

The purpose of mathematical models such as QSAR is to assess the variation in properties of a compound group and identify the mathematical relationship between them, if possible [71,72]. The QSAR model is generally limited to the early stages of drug development and primarily used to identify and exclude molecules of limited permeability [58,59]. Although this model can rapidly assess the relationship between physiochemical properties and biopharmaceutical processes, it is strictly limited by the data that is available from *in vitro* and *in vivo* studies, hence its limitation of use [72].

Alternatively, oral PBPK models are becoming increasingly popular; these dynamic, mathematical models provide a robust *in vitro – in vivo* prediction and are highly sought after by various pharmaceutical companies at various stages of drug development [26,55,58]. There are a variety of PBPK models currently available, all are of high value in the selection and optimization of drug form and formulation stages of oral drug development [55,59]. The mechanistic nature of PBPK models is a factor that makes them much more complex than QSAR but allows them to incorporate physiological processes of the gut [55]. PBPK models are built up using data obtained from pre-clinical *in vitro* data and data obtained from *in vivo* studies [26,55]. This allows the comparison, and potential validation, of *in vitro* studies to *in vivo* studies [26,55]. It can lead to the confirmation of a successful IVIVC between *in vitro* model to its *in vivo* situation; even further it can confirm the correlation between an animal model and a human *in vivo* study [55]. *In silico* approaches allow alternative models to compete with *in vivo* studies and acts as a tool to identify their compatibility [55].

The development and optimization of reliable oral PBPK models have been relatively recent. Such models have evidently had an impact on the development of *in situ* and *in vitro* models that were previously hindered by doubt and speculation over their relevance to the *in vivo* situation. There are ample oral drug absorption studies performed on animal subjects which incorporate MSI but this is not, however, reflected with GI models. Therefore, the advancements in oral PBPK models that have allowed the *in vivo* relevance of alternative GI models to be assessed may be a factor contributing to the lack of advancements in combining GI models with analytical methods such as MSI. The continual development and improvements made to *in silico* models as discussed have inevitably allowed focus to shift to evaluating and optimizing alternative GI models, such as *in vitro*, to be more relevant to the *in vivo* situation. Hence, providing a potential explanation for the delay in moving to more advanced and novel analytical methods like QMSI which were successfully showcased by Russo *et al*. [50], in dermal drug absorption studies, as previously discussed.

#### 3.1.3. In vitro methods

*In-vitro* methods are defined as experiments that take place outside of a living organism. The term, *in-vitro* is a broad term which can include either cultured cells or *ex-vivo* tissue [66]. Regardless of whether cells or tissue are used for drug absorption studies, the specimen is placed in a diffusion cell and intestinal absorption is assessed. As a collective, *in vitro* studies have a number of advantages in comparison to those conducted *in vivo*; these include a significant reduction in the amount of drug required for the experiment and the avoidance of complex surgery and animal maintenance [59]. Traditional *in vitro* methods, whether *ex vivo* tissue or cultured cells, are disadvantaged by the fact that natural physiological factors are not involved in the data interpretation process; for instance, gastric juices [63,67].

For oral drug studies, the cultured cells that are typically used to study and assess drug absorption include Madin-Darby canine kidney (MDCK) line and colorectal adenocarcinoma (caco-2) cells, with the latter being considered a gold standard as well as the most established [54,66]. Each cell line has its own individual advantages and limitations; for instance, caco-2 cells can differentiate and express some efflux transporters, however, the lack of drug metabolizing P450 enzymes makes it unsuitable for the study of some drug classes [59]. A frequently reported analytical method used in combination with caco-2 cells for permeation studies is LC-MS/MS. Although, historically radiolabelling techniques were used. Lu *et al*. [73], recently reported the validation of an ultra-high-performance liquid chromatography-tandem mass spectrometry (UPLC-MS/MS) to quantify the amount and consequent intestinal permeability of the anticholinergic drug, Trihexyphenidyl hydrochloride, in caco-2 cells. The study successfully validated the method and in addition, gained information on the transport mechanism of that specific drug.

The use of cultured cells for *in vitro* experiments significantly reduces animal usage and allows many variables to be explored within one experiment; *ex vivo* tissue used for *in vitro* experiments shares this benefit although the number of animals is not as greatly reduced [59]. *Ex vivo* tissue models utilize tissue that has been removed from a live subject although, the ability to explore numerous variables at once reduces the number of animal sacrifices required [67]. These models require relatively small amounts of tissue and so, allow multiple experiments per animal sacrifice [59]. It would, therefore, be in the best interest of those especially focused on reducing animal testing to analyze *in vitro* models, with focus on *ex vivo* tissue to utilize techniques such as MSI. The ability of MSI to image several analytes including endogenous compounds in addition to drug molecules would further minimize use of animal subjects.

For oral drug studies, *ex vivo* tissue models include the everted gut sac technique and the Ussing chamber [59,74]. The everted gut sac technique is limited to the use of animal intestines, whereby the intestine is removed from the sacrificed animal and everted over a glass rod to create an everted sac [59,67]. Culture medium fills the inside of the sac which is then sat in a vessel filled with the drug solution, allowing intestinal drug transport to be simulated [59]. The amount of drug that has accumulated inside the sac is measured and allows drug absorption to be quantitively assessed [59]. A major advantage of this technique is that it is an analytically simple method for the study of drug absorption as well as the analysis of absorption boosting excipients [66]. As with caco-2 cells, there is a clear abundance of studies that combine the everted gut sac technique with LC-MS/MS for quantitative analysis. The combination of UPLC-MS/MS with the everted gut sac model has shown promise in the drug discovery phases with Gao *et al*. [75], reporting the identification and quantification of bioactive ingredients within Traditional Chinese Medicine (TCM). In this instance, the TCM was atractylode which is a dried root that is crudely extracted and orally taken in the form of a herbal remedy. The UPLC-MS/MS method was optimized for detection and quantification of known atractylode indicators; this method was then used to quantify these indicators within the everted gut model of a rat [75].

Unlike the Ussing chamber, there are no known publications that report the combination of the everted gut sac technique with MALDI-MSI. The reason for this is unknown, as the process needed to prepare a gut sac for MALDI-MSI would be indistinguishable from that for the tissue used in an Ussing chamber model. The Ussing chamber model works on a similar principle to the everted gut sac technique, however, the intestine is used in a sheet format rather than a sac and is mounted between two compartments [59,65]. The amount of drug that has moved from one compartment, through the intestinal tissue to the other compartment is then quantified, calculating the apparent permeability coefficients (P_app_) as with cultured cell experiments [58,65]. In addition to drug absorption and permeation, bidirectional drug transport can also be studied using the Ussing chamber [59,76]. A major benefit of using *ex vivo* tissues *in vitro* is that the apical mucus layer remains, which makes the model more relevant to the *in vivo* situation than cultured cells which lack a mucus layer [67]. Although, the partial removal of the serosal muscle layer from epithelial tissue to complete direct permeation studies is reportedly a difficult task to perform [65,76].

Similarly, to the other *in vitro* methods, there are a vast amount of studies that utilize LC-MS/MS to quantify drug permeation and transport through the intestinal membrane. In addition to this, there are published studies reporting MALDI-MSI analysis of tissue from an Ussing chamber. Tanaka *et al*. [77], recently reported the successful use of MALDI-MSI to visualize oral anti-atherosclerotic dipeptides, Trp-His and His-Trp, rat intestinal membrane that had been exposed to the dipeptides in the Ussing chamber experiments. The MALDI-MSI analysis provided valuable information that one of the dipeptides faced hydrolysis at the brush border membranes, which would have otherwise been unknown. To gain quantitative information as would be acquired using LC-MS/MS, QMSI is a very novel technique that could easily be implemented to studies such as those discussed here. QMSI overcomes the quantification issues usually caused by ion suppression effects by spotting a series of standards to create a calibration graph. This would allow the visualization of drug distribution in addition to quantitative information on the drug within the tissue as well as the amount that had traveled through the tissue.

Of all the gastrointestinal models discussed, the majority have adopted mass spectrometry for quantitative analysis, namely LC-MS/MS. Models utilizing *ex vivo* tissue have been the main source of MSI publications which have reported the successful mapping of oral drug distribution within tissue. In spite of this, drug permeation and absorption studies appear to fall behind in terms of advancements in mass spectrometry. With the previously discussed study conducted by Russo *et al*. [50], the potential to quantify drugs on an LSE tissue using quantitative MALDI-MSI has been clearly showcased. The study goes on to confirm the accuracy of their quantitative findings by using an LC-MS/MS method for validation. Although MSI has demonstrated the combination of the quantitative abilities of LC-MS/MS and imaging capabilities of PET, it has still not been used in combination with any of the gastrointestinal models previously discussed for oral drug studies.

### 3.2. Microfluidic systems

Similar to the *in vitro* model of the GI tract, microfluidic systems have been developed to recreate the microenvironments of many *in vivo* tissues. Although technically microfluidic systems mostly contain 2D cell cultures, the complex structure is designed to recreate the multiscale architecture and tissue-tissue interfaces that are crucial for organs and tissues to function. Otherwise known as ‘organ-on-a-chip’ the purpose of this system is not to build a whole living organ within a representative native environment, but rather to synthesize minimal functional units that recapitulate tissue and organ level processes. The benefits of using the microfluidic system facilitate the generation of microscale dimensions and volumes that are similar to those typically found in biological systems [78]. The microfluidic device can be designed in multiple ways depending on the complexity of the tissue that is modeled. The basic concept of the system is that the cells are plated within patterns on a chip that is coated with biocompatible materials such as polymer substrates. These materials allow for the passage of nutrients from the microchannels that provide a continuous flow of fluid. Unlike 2D cell cultures and 3D models, including spheroids and organoids, which are typically grown and treated sitting within a well of media. The continuous flow allows for the manipulation of the chemical gradients for cell survival and function over a long time point. This also enables the treatment of drugs in a more representative manner such as recapitulating the oral or IV injection administration route. In more complex designs, numerous microchannels are connected by different porous membranes for different cell types. This constructs the interfaces between different tissue types to recreate a model of the human body. The many attributes of microfluidic systems identify this method of culture as one of the most advanced 3D tissue-engineered models.

An extensive range of research has already utilized these microfluidic systems with mass spectrometry techniques in pharmacological studies. Santbergen *et al*. [79] designed an on-line UPLC-MS technique coupled to a ‘gut-on-a-chip’ model fabricated with a co-culture of colonic adenocarcinoma cell lines, Caco-2 and HT29-MTX. The dynamic system had switching valves to measure the apical and basolateral sides of the *in vitro* model, allowing for permeability analysis of the oral drugs, verapamil, and granisetron. Qualitative and quantitative analysis of the anticancer drug, genistein was demonstrated by Chen *et al*. [80], by developing a stable isotope labeling assisted microfluidic chip electrospray (ESI)-MS platform. The device cultivated MCF-7 breast cancer cells with the tumor growth inhibitor to study cell metabolism, and subsequently calculated the concentration of the eluting drug to determine drug absorption.

Typically, imaging organ-on-chip cultures is performed by either fluorescence or optical microscopy. Microfluidic devices may be amenable to MSI, however there is no literature to date that has utilized this approach. This simply could be due to the fact the microfluidic system is much more complex and lacks accessibility to image these cells. In addition, the sample size of the cultures in a microfluidic system is very small. This is a limitation for multiple reasons, including the inability to reproduce the spatial heterogeneity found in larger 3D models such as organoids. In addition to the spatial resolution challenges of MSI; although, the developments in spatial resolution technology hold potential. Air-liquid interface MSI methods such as DESI or LESA may be better suited with microfluidic devices as for MALDI-MSI required sample preparations and the laser desorption technique could impact the biological composition.

An unconventional approach, however, has interfaced a microfluidic device with MALDI-MSI. Jo *et al*. [81] stimulated neurons cultured onto a microfluidic system and collected the neuronal release on a functionalized surface that is compatible for direct MALDI-MSI analysis. An estimated amount of neuropeptides released including acidic peptide and α-BCP, was calculated by imaging the distance the peptides flowed through the measured channels of the functionalized surface. The group further adapted the MALDI-MSI method to achieve improved accuracy and precision [82]. This method utilized the abilities of MALDI-MSI to image the spatial distribution of the peptides as a measurement tool. Although the study did not directly analyze the spatial integrity of the neurons in culture, the group utilized the MSI method for a novel approach that still examined biological behavior.

**Figure 3.**
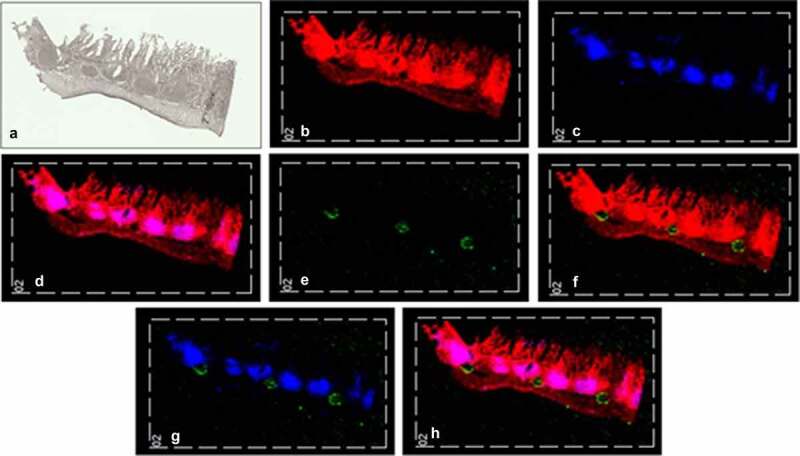
*Ex vivo* small intestine tissue from a Quasi Vivo 600 Liquid-Liquid Interphase *in vitro* system. The small intestinal tissue, with the apical layer facing upwards, was treated with 0.5 mg/mL Atorvastatin over a 6-hour period to investigate drug absorption. (a) A scanned image of the intestinal tissue section taken using a Super Coolscan 5000 ED Film Scanner. (b) A MALDI-MS image showing cholesterol [Chol+H-H_2_O]^+^ at *m/z* 369 in red. (c) A MALDI- MS image showing the Peyer’s patches at *m/z* 389 in blue. (d) A MALDI-MS image showing cholesterol [Chol+H-H_2_O]^+^ at *m/z* 369 in red and Peyer’s patches in blue; overlapping ions are shown in pink. (e) A MALDI-MS image showing the sodium adduct of atorvastatin at *m/z* 581 in green. (f) A MALDI-MS image showing the sodium adduct of atorvastatin at *m/z* 581 in green and cholesterol [Chol+H-H_2_O]^+^ at *m/z* 369 in red. (g) A MALDI-MS image showing sodium adduct of atorvastatin (*m/z* 581) in green and Peyer’s patches (*m/z* 389) in blue. (g) A MALDI-MS image showing the sodium adduct of atorvastatin at *m/z* 581 in green, cholesterol [Chol+H-H_2_O]^+^ at *m/z* 369 in red and Peyer’s patches in blue; overlapping blue and red ions are shown in pink [Credit: C. Spencer, data not published]

As demonstrated by the extensive range of studies, conducted to date, MSI has proven strength in the investigation of pharmacological activity in 3D cell culture models. With the expanding surge of microfluidic systems for the study of therapeutics and the biological response, it can be contemplated that there is a high possibility the MSI-microfluidics approach will be exploited within the foreseeable future. This is especially true when the recent advances in microfluidics to further enhance the complexity of the 3D culture systems are considered. For example, macrofluidic systems or ‘organoid-on-a-chip’ platforms have been engineered to combine the complexity of microfluidic devices with *ex vivo* tissues or organoids, to replicate the *in vivo* microenvironments for patient-derived cultures [83]. For instance, our group has developed a macrofluidics device cultivating an *ex vivo* small intestine tissue on a Quasi Vivo 600 Liquid-Liquid Interphase *in vitro* system to model the GI tract. After treatment, the *ex vivo* tissue was removed from the fluidics system, snap frozen, and cryo-sectioned prior to preparing the sample with matrix by sublimation. MALDI-MSI analysis was able to detect cholesterol [Chol+H-H_2_O]^+^ at *m/z* 369, observed throughout the tissue section (Figure 3b), and lymphatic tissue known as Peyer’s patches at *m/z* 389 identifying the substructures within the small intestine (Figure 3c). A MALDI-MS image showed the sodium adduct of the oral drug, atorvastatin at *m/z* 581 within the apical side of tissue after a 6-hour incubation (Figure 3d). As the distribution of atorvastatin is localized outside of the Peyer’s patches, it indicated an absorption by passive diffusion (Figure 3g). Not only does this demonstrate advanced manipulation of *ex vivo* tissue, but it also allows for even more accurate drug analysis than previously mentioned 3D models such as spheroids or organoid cultures by MALDI-MSI. These advancements in 3D culture models can offer an ideal testing platform in drug developmental studies, which also hold potential to be exploited for analysis by MSI.

## 4. Conclusions

The applications described here that combine MSI with 3D cell culture models are just a small proportion of the published applications that are currently available. To date MALDI-MSI is the most widely used MSI technique for the study of 3D cell culture models. However, MALDI-MSI has well-documented limitations for the study of pharmaceuticals, in particular in the range of compounds that it is applicable to. These limitations do not apply to some of the other MSI techniques namely DESI-MSI and the recently developed MALDI-2 technique [84]. It would be therefore be expected that applications of MSI techniques other than MALDI-MSI will increase over the next period.

It is apparent from the advances made in dermal drug absorption studies that there is a clear opportunity for quantitative MSI (however it is performed) to be used in combination with gastrointestinal models in a similar manner for the study of oral drug absorption. With the demonstrated ability of MSI to detect endogenous compounds such as proteins, a potential future area of excitement would be to combine quantitative MSI with other imaging techniques to study drug absorption within a gastrointestinal macrofluidic system.

## 5. Expert opinion

Mass spectrometry imaging (MSI) is a powerful technique that can map a vast range of molecules simultaneously in tissues without the addition of labels. Modern instruments can measure at single micron pixel size and at accurately and precisely quantify drugs ng mm^−2^ (of tissue area) sensitivity. For the pharmaceutical industry this gives the ability to study and distinguish the distribution of the parent drug and its metabolites whilst simultaneously examining endogenous compounds that may be biomarkers of effect. It has been demonstrated in numerous studies that MSI can provide insights into the efficacy and safety of new drugs when used for both in vitro and in vivo preclinical drug trials. However, with regards to early-stage preclinical studies carried out using animals, there are increasing concerns about both the validity and morality of using animals in drug development experiments for drugs intended for humans. To address these concerns, there is interest in the use of 3D cell cultures, organoids, and engineered tissues produced using human cells for such experiments. The combination of MSI and 3D cell cultures is emerging as a promising tool in early-stage drug development experiment. There is an increasing body of work appearing in the literature describing the combination of MSI with 3D cell culture and engineered tissue. This particularly applies to drug distribution and action studies in experiments involving the use of tumor spheroids and tissue-engineered skin. Methodology for the quantitative study of drug distribution, drug metabolism, efficacy, and toxicity have been successfully demonstrated using these sample types. However, whilst such studies are of a great deal of interest, the most common administration route for pharmaceutical drugs is via oral delivery. An area of intensive study in pharma is drug absorption in the GI tract particularly via the small intestine. At present when such studies are performed in vitro using static 2D cell culture using permeation chamber type devices are employed. The use of MSI in combination with 3D models of the GI tract is an area that has been little explored to date. The primary barrier to development in this area has been the lack of suitable models. However, reports of GI tract modeling using organoids have recently appeared in the literature and this can be incorporated into static systems. However, using 3D tissue models in combination with microfluidic systems creates the opportunity to generate minimal functional units that recapitulate tissue and organ level processes. Preliminary work has been carried out using microfluidic systems in combination with excised animal tissue and MSI in our own laboratory. A real opportunity for valuable future work lies in successful combination of GI tract models, microfluidic systems, and MSI and it would be expected that over the next ten years this combination; tissue model, microfluidic system, and MSI will find more and more application over and above the GI tract. This will both generate data more applicable to humans than animals or 2D cell culture and contribute to the 3Rs (reduction, refinement, and replacement) agenda in experiments conducted in pre-clinical drug development.

## Article Highlights

- There is high demand for *in vitro* models that accurately replicate the *in vivo* environment and responds to the societal requirements to reduce animal numbers in research
- A variety of 3D cell culture models have been developed to meet this demand and have found use in studies of drug distribution, efficacy and toxicity, patient-derived treatments, and biological crosstalk.
- The multiplex nature of mass spectrometry imaging creates opportunities for the detailed analysis of 3D cell culture models.
- The main 3D cell culture models: spheroids, organoids analyzed by mass spectrometry to date are discussed.
- The potential of 3D tissue-engineered systems of the GI tract in combination with microfluidics and mass spectrometry imaging is discussed as an exciting future application of the technology.
